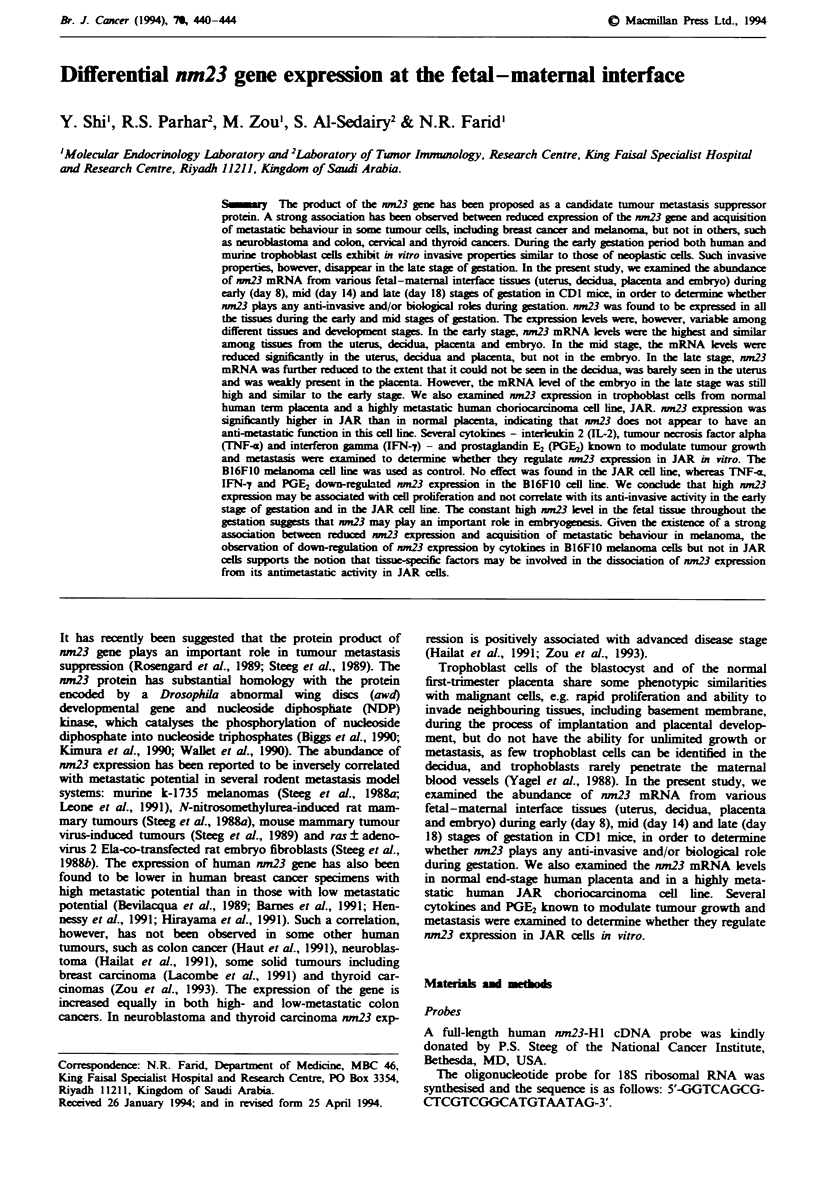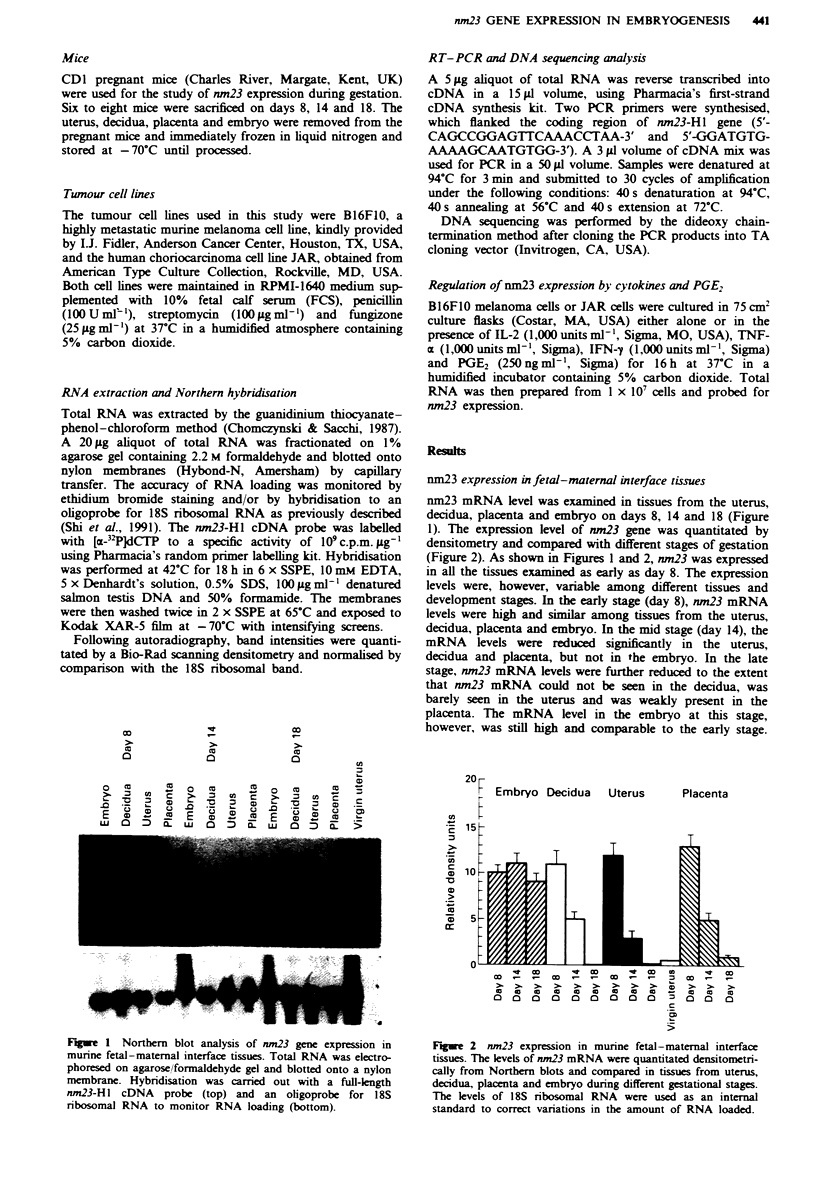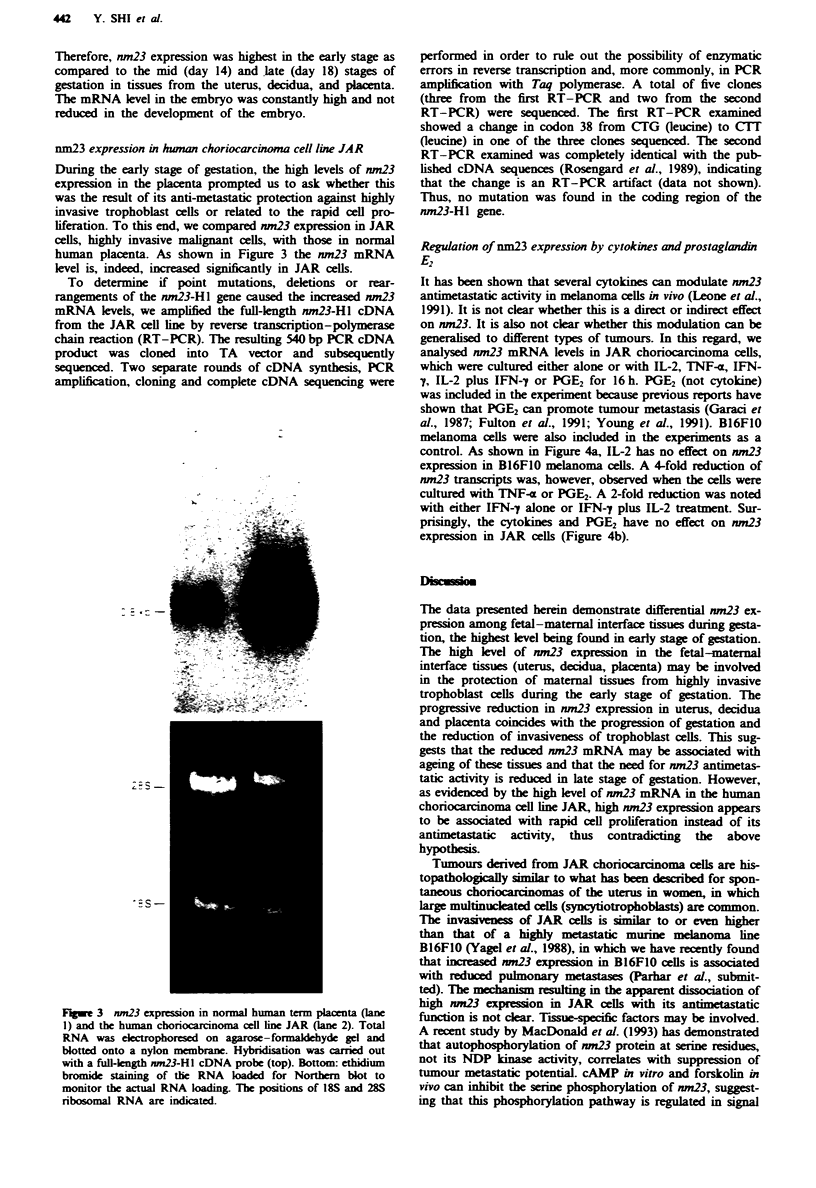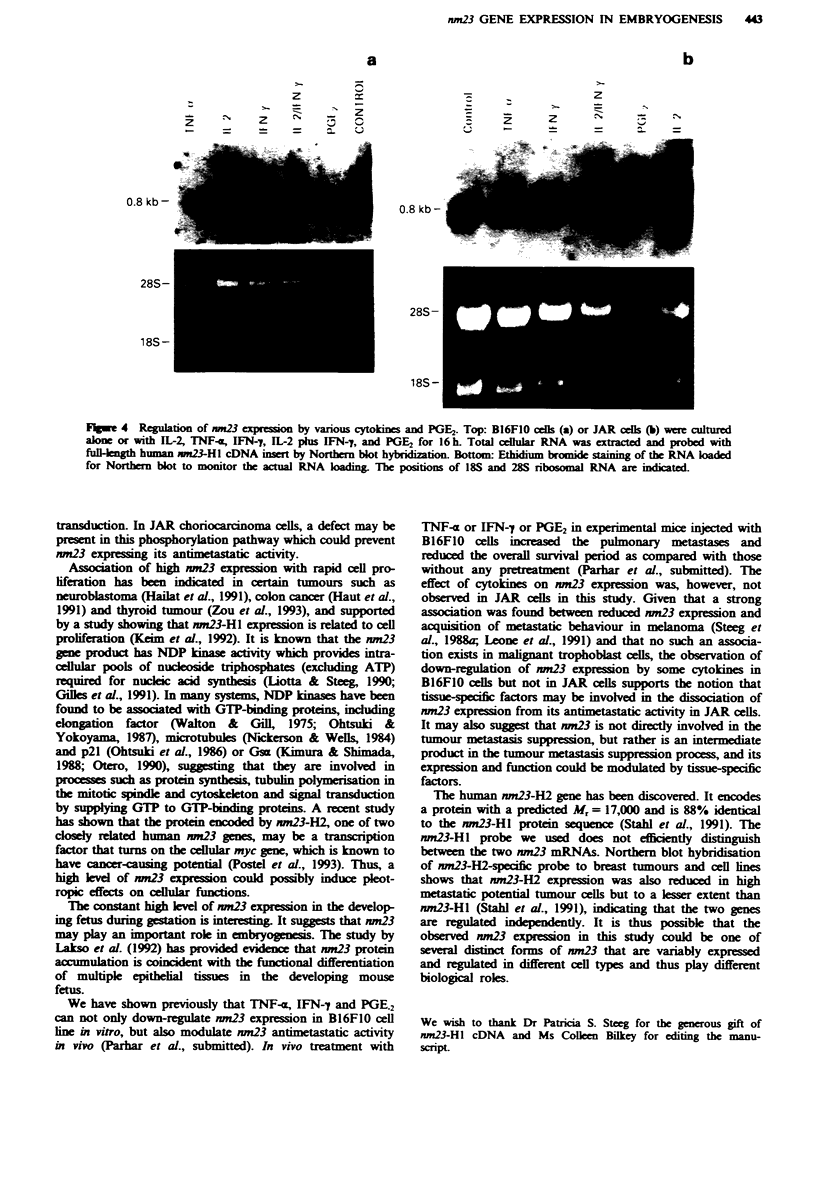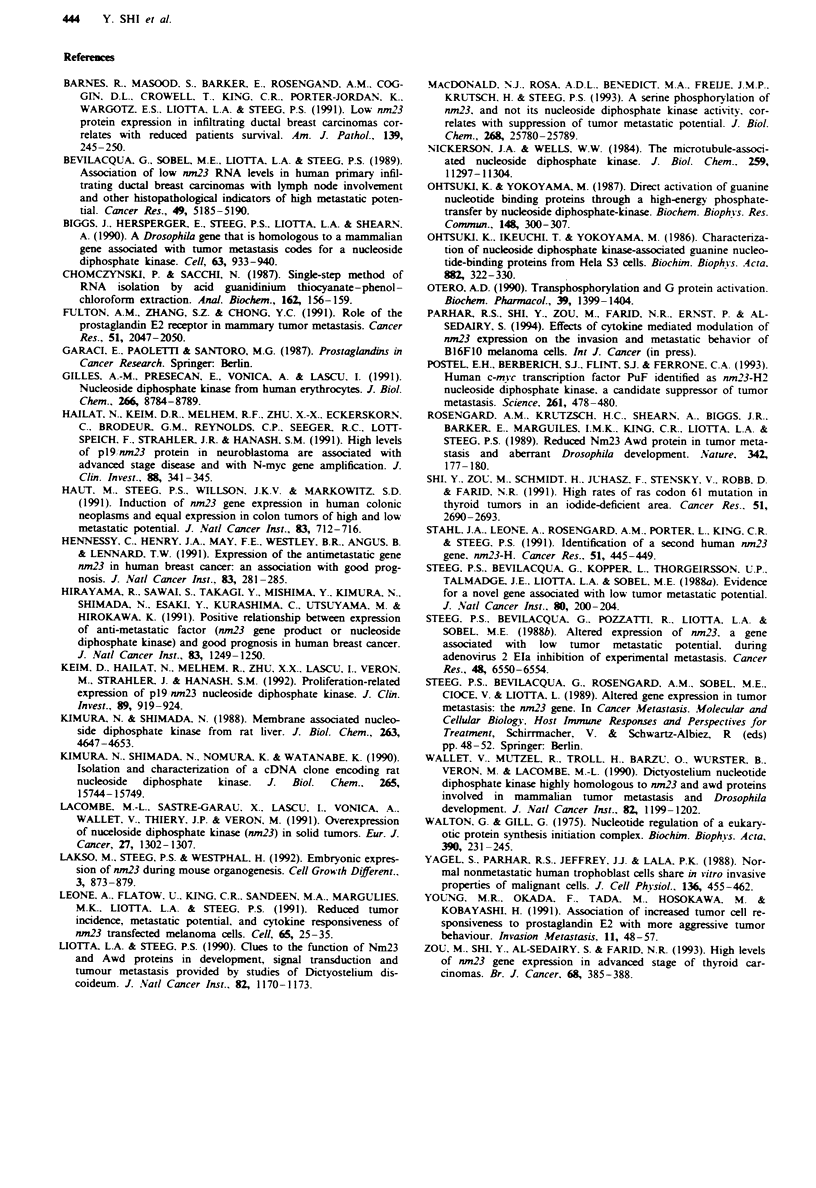# Differential nm23 gene expression at the fetal-maternal interface.

**DOI:** 10.1038/bjc.1994.324

**Published:** 1994-09

**Authors:** Y. Shi, R. S. Parhar, M. Zou, S. al-Sedairy, N. R. Farid

**Affiliations:** Molecular Endocrinology Laboratory, King Faisal Specialist Hospital and Research Centre, Riyadh, Kingdom of Saudi Arabia.

## Abstract

**Images:**


Br. J. Cancer (1994), 70, 440-444 © Macmillan Press Ltd., 1994

### Differential nm23 gene expression at the fetal-maternal interface

Y. Shi[1], R.S. Parhar[2], M. Zou[1], S. Al-Sedairy[2] & N.R. Farid[1]

[1]*Molecular Endocrinology Laboratory and* [2]*Laboratory of Tumor Immunology, Research Centre, King Faisal Specialist Hospital and Research Centre, Riyadh 11211, Kingdom of Saudi Arabia.*

**Summary** The product of the *nm23* gene has been proposed as a candidate tumour metastasis suppressor protein. A strong association has been observed between reduced expression of the *nm23* gene and acquisition of metastatic behaviour in some tumour cells, including breast cancer and melanoma, but not in others, such as neuroblastoma and colon, cervical and thyroid cancers. During the early gestation period both human and murine trophoblast cells exhibit *in vitro* invasive properties similar to those of neoplastic cells. Such invasive properties, however, disappear in the late stage of gestation. In the present study, we examined the abundance of *nm23* mRNA from various fetal-maternal interface tissues (uterus, decidua, placenta and embryo) during early (day 8), mid (day 14) and late (day 18) stages of gestation in CD1 mice, in order to determine whether *nm23* plays any anti-invasive and/or biological roles during gestation. *nm23* was found to be expressed in all the tissues during the early and mid stages of gestation. The expression levels were, however, variable among different tissues and development stages. In the early stage, *nm23* mRNA levels were the highest and similar among tissues from the uterus, decidua, placenta and embryo. In the mid stage, the mRNA levels were reduced significantly in the uterus, decidua and placenta, but not in the embryo. In the late stage, *nm23* mRNA was further reduced to the extent that it could not be seen in the decidua, was barely seen in the uterus and was weakly present in the placenta. However, the mRNA level of the embryo in the late stage was still high and similar to the early stage. We also examined *nm23* expression in trophoblast cells from normal human term placenta and a highly metastatic human choriocarcinoma cell line, JAR. *nm23* expression was significantly higher in JAR than in normal placenta, indicating that *nm23* does not appear to have an anti-metastatic function in this cell line. Several cytokines - interleukin 2 (IL-2), tumour necrosis factor alpha (TNF-$\alpha$) and interferon gamma (IFN-$\gamma$) - and prostaglandin $E_2$ ($PGE_2$) known to modulate tumour growth and metastasis were examined to determine whether they regulate *nm23* expression in JAR *in vitro*. The B16F10 melanoma cell line was used as control. No effect was found in the JAR cell line, whereas TNF-$\alpha$, IFN-$\gamma$ and $PGE_2$ down-regulated *nm23* expression in the B16F10 cell line. We conclude that high *nm23* expression may be associated with cell proliferation and not correlate with its anti-invasive activity in the early stage of gestation and in the JAR cell line. The constant high *nm23* level in the fetal tissue throughout the gestation suggests that *nm23* may play an important role in embryogenesis. Given the existence of a strong association between reduced *nm23* expression and acquisition of metastatic behaviour in melanoma, the observation of down-regulation of *nm23* expression by cytokines in B16F10 melanoma cells but not in JAR cells supports the notion that tissue-specific factors may be involved in the dissociation of *nm23* expression from its antimetastatic activity in JAR cells.

It has recently been suggested that the protein product of *nm23* gene plays an important role in tumour metastasis suppression (Rosengard *et al.*, 1989; Steeg *et al.*, 1989). The *nm23* protein has substantial homology with the protein encoded by a *Drosophila* abnormal wing discs (*awd*) developmental gene and nucleoside diphosphate (NDP) kinase, which catalyses the phosphorylation of nucleoside diphosphate into nucleoside triphosphates (Biggs *et al.*, 1990; Kimura *et al.*, 1990; Wallet *et al.*, 1990). The abundance of *nm23* expression has been reported to be inversely correlated with metastatic potential in several rodent metastasis model systems: murine k-1735 melanomas (Steeg *et al.*, 1988*a*; Leone *et al.*, 1991), *N*-nitrosomethylurea-induced rat mammary tumours (Steeg *et al.*, 1988*a*), mouse mammary tumour virus-induced tumours (Steeg *et al.*, 1989) and *ras* ± adenovirus 2 Ela-co-transfected rat embryo fibroblasts (Steeg *et al.*, 1988*b*). The expression of human *nm23* gene has also been found to be lower in human breast cancer specimens with high metastatic potential than in those with low metastatic potential (Bevilacqua *et al.*, 1989; Barnes *et al.*, 1991; Hennessy *et al.*, 1991; Hirayama *et al.*, 1991). Such a correlation, however, has not been observed in some other human tumours, such as colon cancer (Haut *et al.*, 1991), neuroblastoma (Hailat *et al.*, 1991), some solid tumours including breast carcinoma (Lacombe *et al.*, 1991) and thyroid carcinomas (Zou *et al.*, 1993). The expression of the gene is increased equally in both high- and low-metastatic colon cancers. In neuroblastoma and thyroid carcinoma *nm23* expression is positively associated with advanced disease stage (Hailat *et al.*, 1991; Zou *et al.*, 1993).

Trophoblast cells of the blastocyst and of the normal first-trimester placenta share some phenotypic similarities with malignant cells, e.g. rapid proliferation and ability to invade neighbouring tissues, including basement membrane, during the process of implantation and placental development, but do not have the ability for unlimited growth or metastasis, as few trophoblast cells can be identified in the decidua, and trophoblasts rarely penetrate the maternal blood vessels (Yagel *et al.*, 1988). In the present study, we examined the abundance of *nm23* mRNA from various fetal-maternal interface tissues (uterus, decidua, placenta and embryo) during early (day 8), mid (day 14) and late (day 18) stages of gestation in CD1 mice, in order to determine whether *nm23* plays any anti-invasive and/or biological role during gestation. We also examined the *nm23* mRNA levels in normal end-stage human placenta and in a highly metastatic human JAR choriocarcinoma cell line. Several cytokines and $PGE_2$ known to modulate tumour growth and metastasis were examined to determine whether they regulate *nm23* expression in JAR cells *in vitro*.

### Materials and methods

### Probes

A full-length human *nm23*-H1 cDNA probe was kindly donated by P.S. Steeg of the National Cancer Institute, Bethesda, MD, USA.

The oligonucleotide probe for 18S ribosomal RNA was synthesised and the sequence is as follows: 5′-GGTCAGCG-CTCGTCGGCATGTAATAG-3′.

Correspondence: N.R. Farid, Department of Medicine, MBC 46, King Faisal Specialist Hospital and Research Centre, PO Box 3354, Riyadh 11211, Kingdom of Saudi Arabia.
Received 26 January 1994; and in revised form 25 April 1994.

### *Mice*

CD1 pregnant mice (Charles River, Margate, Kent, UK) were used for the study of *nm23* expression during gestation. Six to eight mice were sacrificed on days 8, 14 and 18. The uterus, decidua, placenta and embryo were removed from the pregnant mice and immediately frozen in liquid nitrogen and stored at −70°C until processed.

### *Tumour cell lines*

The tumour cell lines used in this study were B16F10, a highly metastatic murine melanoma cell line, kindly provided by I.J. Fidler, Anderson Cancer Center, Houston, TX, USA, and the human choriocarcinoma cell line JAR, obtained from American Type Culture Collection, Rockville, MD, USA. Both cell lines were maintained in RPMI-1640 medium supplemented with 10% fetal calf serum (FCS), penicillin (100 U $ml^{-1}$), streptomycin (100 $\mu g\ ml^{-1}$) and fungizone (25 $\mu g\ ml^{-1}$) at 37°C in a humidified atmosphere containing 5% carbon dioxide.

### *RNA extraction and Northern hybridisation*

Total RNA was extracted by the guanidinium thiocyanate–phenol–chloroform method (Chomczynski & Sacchi, 1987). A 20 $\mu g$ aliquot of total RNA was fractionated on 1% agarose gel containing 2.2 M formaldehyde and blotted onto nylon membranes (Hybond-N, Amersham) by capillary transfer. The accuracy of RNA loading was monitored by ethidium bromide staining and/or by hybridisation to an oligoprobe for 18S ribosomal RNA as previously described (Shi *et al.*, 1991). The *nm23*-H1 cDNA probe was labelled with [$\alpha$-$^{32}$P]dCTP to a specific activity of $10^9$ c.p.m. $\mu g^{-1}$ using Pharmacia's random primer labelling kit. Hybridisation was performed at 42°C for 18 h in 6 × SSPE, 10 mM EDTA, 5 × Denhardt's solution, 0.5% SDS, 100 $\mu g\ ml^{-1}$ denatured salmon testis DNA and 50% formamide. The membranes were then washed twice in 2 × SSPE at 65°C and exposed to Kodak XAR-5 film at −70°C with intensifying screens.

Following autoradiography, band intensities were quantitated by a Bio-Rad scanning densitometry and normalised by comparison with the 18S ribosomal band.

**Figure 1** Northern blot analysis of *nm23* gene expression in murine fetal–maternal interface tissues. Total RNA was electrophoresed on agarose/formaldehyde gel and blotted onto a nylon membrane. Hybridisation was carried out with a full-length *nm23*-H1 cDNA probe (top) and an oligoprobe for 18S ribosomal RNA to monitor RNA loading (bottom).

### *RT–PCR and DNA sequencing analysis*

A 5 $\mu g$ aliquot of total RNA was reverse transcribed into cDNA in a 15 $\mu l$ volume, using Pharmacia's first-strand cDNA synthesis kit. Two PCR primers were synthesised, which flanked the coding region of *nm23*-H1 gene (5'-CAGCCGGAGTTCAAACCTAA-3' and 5'-GGATGTG-AAAAGCAATGTGG-3'). A 3 $\mu l$ volume of cDNA mix was used for PCR in a 50 $\mu l$ volume. Samples were denatured at 94°C for 3 min and submitted to 30 cycles of amplification under the following conditions: 40 s denaturation at 94°C, 40 s annealing at 56°C and 40 s extension at 72°C.

DNA sequencing was performed by the dideoxy chain-termination method after cloning the PCR products into TA cloning vector (Invitrogen, CA, USA).

### *Regulation of* nm23 *expression by cytokines and $PGE_2$*

B16F10 melanoma cells or JAR cells were cultured in 75 $cm^2$ culture flasks (Costar, MA, USA) either alone or in the presence of IL-2 (1,000 units $ml^{-1}$, Sigma, MO, USA), TNF-$\alpha$ (1,000 units $ml^{-1}$, Sigma), IFN-$\gamma$ (1,000 units $ml^{-1}$, Sigma) and $PGE_2$ (250 ng $ml^{-1}$, Sigma) for 16 h at 37°C in a humidified incubator containing 5% carbon dioxide. Total RNA was then prepared from 1 × $10^7$ cells and probed for *nm23* expression.

### Results

### nm23 *expression in fetal–maternal interface tissues*

nm23 mRNA level was examined in tissues from the uterus, decidua, placenta and embryo on days 8, 14 and 18 (Figure 1). The expression level of *nm23* gene was quantitated by densitometry and compared with different stages of gestation (Figure 2). As shown in Figures 1 and 2, *nm23* was expressed in all the tissues examined as early as day 8. The expression levels were, however, variable among different tissues and development stages. In the early stage (day 8), *nm23* mRNA levels were high and similar among tissues from the uterus, decidua, placenta and embryo. In the mid stage (day 14), the mRNA levels were reduced significantly in the uterus, decidua and placenta, but not in the embryo. In the late stage, *nm23* mRNA levels were further reduced to the extent that *nm23* mRNA could not be seen in the decidua, was barely seen in the uterus and was weakly present in the placenta. The mRNA level in the embryo at this stage, however, was still high and comparable to the early stage.

**Figure 2** *nm23* expression in murine fetal–maternal interface tissues. The levels of *nm23* mRNA were quantitated densitometrically from Northern blots and compared in tissues from uterus, decidua, placenta and embryo during different gestational stages. The levels of 18S ribosomal RNA were used as an internal standard to correct variations in the amount of RNA loaded.

Therefore, *nm23* expression was highest in the early stage as compared to the mid (day 14) and late (day 18) stages of gestation in tissues from the uterus, decidua, and placenta. The mRNA level in the embryo was constantly high and not reduced in the development of the embryo.

### nm23 *expression in human choriocarcinoma cell line JAR*

During the early stage of gestation, the high levels of *nm23* expression in the placenta prompted us to ask whether this was the result of its anti-metastatic protection against highly invasive trophoblast cells or related to the rapid cell proliferation. To this end, we compared *nm23* expression in JAR cells, highly invasive malignant cells, with those in normal human placenta. As shown in Figure 3 the *nm23* mRNA level is, indeed, increased significantly in JAR cells.

To determine if point mutations, deletions or rearrangements of the *nm23*-H1 gene caused the increased *nm23* mRNA levels, we amplified the full-length *nm23*-H1 cDNA from the JAR cell line by reverse transcription-polymerase chain reaction (RT-PCR). The resulting 540 bp PCR cDNA product was cloned into TA vector and subsequently sequenced. Two separate rounds of cDNA synthesis, PCR amplification, cloning and complete cDNA sequencing were performed in order to rule out the possibility of enzymatic errors in reverse transcription and, more commonly, in PCR amplification with *Taq* polymerase. A total of five clones (three from the first RT-PCR and two from the second RT-PCR) were sequenced. The first RT-PCR examined showed a change in codon 38 from CTG (leucine) to CTT (leucine) in one of the three clones sequenced. The second RT-PCR examined was completely identical with the published cDNA sequences (Rosengard *et al.*, 1989), indicating that the change is an RT-PCR artifact (data not shown). Thus, no mutation was found in the coding region of the *nm23*-H1 gene.

**Figure 3** *nm23* expression in normal human term placenta (lane 1) and the human choriocarcinoma cell line JAR (lane 2). Total RNA was electrophoresed on agarose-formaldehyde gel and blotted onto a nylon membrane. Hybridisation was carried out with a full-length *nm23*-H1 cDNA probe (top). Bottom: ethidium bromide staining of the RNA loaded for Northern blot to monitor the actual RNA loading. The positions of 18S and 28S ribosomal RNA are indicated.

### *Regulation of* nm23 *expression by cytokines and prostaglandin* $E_2$

It has been shown that several cytokines can modulate *nm23* antimetastatic activity in melanoma cells *in vivo* (Leone *et al.*, 1991). It is not clear whether this is a direct or indirect effect on *nm23*. It is also not clear whether this modulation can be generalised to different types of tumours. In this regard, we analysed *nm23* mRNA levels in JAR choriocarcinoma cells, which were cultured either alone or with IL-2, TNF-$\alpha$, IFN-$\gamma$, IL-2 plus IFN-$\gamma$ or $PGE_2$ for 16 h. $PGE_2$ (not cytokine) was included in the experiment because previous reports have shown that $PGE_2$ can promote tumour metastasis (Garaci *et al.*, 1987; Fulton *et al.*, 1991; Young *et al.*, 1991). B16F10 melanoma cells were also included in the experiments as a control. As shown in Figure 4a, IL-2 has no effect on *nm23* expression in B16F10 melanoma cells. A 4-fold reduction of *nm23* transcripts was, however, observed when the cells were cultured with TNF-$\alpha$ or $PGE_2$. A 2-fold reduction was noted with either IFN-$\gamma$ alone or IFN-$\gamma$ plus IL-2 treatment. Surprisingly, the cytokines and $PGE_2$ have no effect on *nm23* expression in JAR cells (Figure 4b).

### Discussion

The data presented herein demonstrate differential *nm23* expression among fetal-maternal interface tissues during gestation, the highest level being found in early stage of gestation. The high level of *nm23* expression in the fetal-maternal interface tissues (uterus, decidua, placenta) may be involved in the protection of maternal tissues from highly invasive trophoblast cells during the early stage of gestation. The progressive reduction in *nm23* expression in uterus, decidua and placenta coincides with the progression of gestation and the reduction of invasiveness of trophoblast cells. This suggests that the reduced *nm23* mRNA may be associated with ageing of these tissues and that the need for *nm23* antimetastatic activity is reduced in late stage of gestation. However, as evidenced by the high level of *nm23* mRNA in the human choriocarcinoma cell line JAR, high *nm23* expression appears to be associated with rapid cell proliferation instead of its antimetastatic activity, thus contradicting the above hypothesis.

Tumours derived from JAR choriocarcinoma cells are histopathologically similar to what has been described for spontaneous choriocarcinomas of the uterus in women, in which large multinucleated cells (syncytiotrophoblasts) are common. The invasiveness of JAR cells is similar to or even higher than that of a highly metastatic murine melanoma line B16F10 (Yagel *et al.*, 1988), in which we have recently found that increased *nm23* expression in B16F10 cells is associated with reduced pulmonary metastases (Parhar *et al.*, submitted). The mechanism resulting in the apparent dissociation of high *nm23* expression in JAR cells with its antimetastatic function is not clear. Tissue-specific factors may be involved. A recent study by MacDonald *et al.* (1993) has demonstrated that autophosphorylation of *nm23* protein at serine residues, not its NDP kinase activity, correlates with suppression of tumour metastatic potential. cAMP *in vitro* and forskolin *in vivo* can inhibit the serine phosphorylation of *nm23*, suggesting that this phosphorylation pathway is regulated in signal

**Figure 4** Regulation of *nm23* expression by various cytokines and $PGE_2$. Top: B16F10 cells (**a**) or JAR cells (**b**) were cultured alone or with IL-2, TNF-α, IFN-γ, IL-2 plus IFN-γ, and $PGE_2$ for 16 h. Total cellular RNA was extracted and probed with full-length human *nm23*-H1 cDNA insert by Northern blot hybridization. Bottom: Ethidium bromide staining of the RNA loaded for Northern blot to monitor the actual RNA loading. The positions of 18S and 28S ribosomal RNA are indicated.

transduction. In JAR choriocarcinoma cells, a defect may be present in this phosphorylation pathway which could prevent *nm23* expressing its antimetastatic activity.

Association of high *nm23* expression with rapid cell proliferation has been indicated in certain tumours such as neuroblastoma (Hailat *et al.*, 1991), colon cancer (Haut *et al.*, 1991) and thyroid tumour (Zou *et al.*, 1993), and supported by a study showing that *nm23*-H1 expression is related to cell proliferation (Keim *et al.*, 1992). It is known that the *nm23* gene product has NDP kinase activity which provides intracellular pools of nucleoside triphosphates (excluding ATP) required for nucleic acid synthesis (Liotta & Steeg, 1990; Gilles *et al.*, 1991). In many systems, NDP kinases have been found to be associated with GTP-binding proteins, including elongation factor (Walton & Gill, 1975; Ohtsuki & Yokoyama, 1987), microtubules (Nickerson & Wells, 1984) and p21 (Ohtsuki *et al.*, 1986) or Gsα (Kimura & Shimada, 1988; Otero, 1990), suggesting that they are involved in processes such as protein synthesis, tubulin polymerisation in the mitotic spindle and cytoskeleton and signal transduction by supplying GTP to GTP-binding proteins. A recent study has shown that the protein encoded by *nm23*-H2, one of two closely related human *nm23* genes, may be a transcription factor that turns on the cellular *myc* gene, which is known to have cancer-causing potential (Postel *et al.*, 1993). Thus, a high level of *nm23* expression could possibly induce pleotropic effects on cellular functions.

The constant high level of *nm23* expression in the developing fetus during gestation is interesting. It suggests that *nm23* may play an important role in embryogenesis. The study by Lakso *et al.* (1992) has provided evidence that *nm23* protein accumulation is coincident with the functional differentiation of multiple epithelial tissues in the developing mouse fetus.

We have shown previously that TNF-α, IFN-γ and $PGE_2$ can not only down-regulate *nm23* expression in B16F10 cell line *in vitro*, but also modulate *nm23* antimetastatic activity *in vivo* (Parhar *et al.*, submitted). *In vivo* treatment with TNF-α or IFN-γ or $PGE_2$ in experimental mice injected with B16F10 cells increased the pulmonary metastases and reduced the overall survival period as compared with those without any pretreatment (Parhar *et al.*, submitted). The effect of cytokines on *nm23* expression was, however, not observed in JAR cells in this study. Given that a strong association was found between reduced *nm23* expression and acquisition of metastatic behaviour in melanoma (Steeg *et al.*, 1988*a*; Leone *et al.*, 1991) and that no such an association exists in malignant trophoblast cells, the observation of down-regulation of *nm23* expression by some cytokines in B16F10 cells but not in JAR cells supports the notion that tissue-specific factors may be involved in the dissociation of *nm23* expression from its antimetastatic activity in JAR cells. It may also suggest that *nm23* is not directly involved in the tumour metastasis suppression, but rather is an intermediate product in the tumour metastasis suppression process, and its expression and function could be modulated by tissue-specific factors.

The human *nm23*-H2 gene has been discovered. It encodes a protein with a predicted $M_r$ = 17,000 and is 88% identical to the *nm23*-H1 protein sequence (Stahl *et al.*, 1991). The *nm23*-H1 probe we used does not efficiently distinguish between the two *nm23* mRNAs. Northern blot hybridisation of *nm23*-H2-specific probe to breast tumours and cell lines shows that *nm23*-H2 expression was also reduced in high metastatic potential tumour cells but to a lesser extent than *nm23*-H1 (Stahl *et al.*, 1991), indicating that the two genes are regulated independently. It is thus possible that the observed *nm23* expression in this study could be one of several distinct forms of *nm23* that are variably expressed and regulated in different cell types and thus play different biological roles.

We wish to thank Dr Patricia S. Steeg for the generous gift of *nm23*-H1 cDNA and Ms Colleen Bilkey for editing the manuscript.

### References

BARNES, R., MASOOD, S., BARKER, E., ROSENGAND, A.M., COGGIN, D.L., CROWELL, T., KING, C.R., PORTER-JORDAN, K., WARGOTZ, E.S., LIOTTA, L.A. & STEEG, P.S. (1991). Low *nm23* protein expression in infiltrating ductal breast carcinomas correlates with reduced patients survival. *Am. J. Pathol.*, **139**, 245-250.

BEVILACQUA, G., SOBEL, M.E., LIOTTA, L.A. & STEEG, P.S. (1989). Association of low *nm23* RNA levels in human primary infiltrating ductal breast carcinomas with lymph node involvement and other histopathological indicators of high metastatic potential. *Cancer Res.*, **49**, 5185-5190.

BIGGS, J., HERSPERGER, E., STEEG, P.S., LIOTTA, L.A. & SHEARN, A. (1990). A *Drosophila* gene that is homologous to a mammalian gene associated with tumor metastasis codes for a nucleoside diphosphate kinase. *Cell*, **63**, 933-940.

CHOMCZYNSKI, P. & SACCHI, N. (1987). Single-step method of RNA isolation by acid guanidinium thiocyanate-phenol-chloroform extraction. *Anal. Biochem.*, **162**, 156-159.

FULTON, A.M., ZHANG, S.Z. & CHONG, Y.C. (1991). Role of the prostaglandin E2 receptor in mammary tumor metastasis. *Cancer Res.*, **51**, 2047-2050.

GARACI, E., PAOLETTI & SANTORO, M.G. (1987). *Prostaglandins in Cancer Research*. Springer: Berlin.

GILLES, A.-M., PRESECAN, E., VONICA, A. & LASCU, I. (1991). Nucleoside diphosphate kinase from human erythrocytes. *J. Biol. Chem.*, **266**, 8784-8789.

HAILAT, N., KEIM, D.R., MELHEM, R.F., ZHU, X.-X., ECKERSKORN, C., BRODEUR, G.M., REYNOLDS, C.P., SEEGER, R.C., LOTTSPEICH, F., STRAHLER, J.R. & HANASH, S.M. (1991). High levels of p19/*nm23* protein in neuroblastoma are associated with advanced stage disease and with N-myc gene amplification. *J. Clin. Invest.*, **88**, 341-345.

HAUT, M., STEEG, P.S., WILLSON, J.K.V. & MARKOWITZ, S.D. (1991). Induction of *nm23* gene expression in human colonic neoplasms and equal expression in colon tumors of high and low metastatic potential. *J. Natl Cancer Inst.*, **83**, 712-716.

HENNESSY, C., HENRY, J.A., MAY, F.E., WESTLEY, B.R., ANGUS, B. & LENNARD, T.W. (1991). Expression of the antimetastatic gene *nm23* in human breast cancer: an association with good prognosis. *J. Natl Cancer Inst.*, **83**, 281-285.

HIRAYAMA, R., SAWAI, S., TAKAGI, Y., MISHIMA, Y., KIMURA, N., SHIMADA, N., ESAKI, Y., KURASHIMA, C., UTSUYAMA, M. & HIROKAWA, K. (1991). Positive relationship between expression of anti-metastatic factor (*nm23* gene product or nucleoside diphosphate kinase) and good prognosis in human breast cancer. *J. Natl Cancer Inst.*, **83**, 1249-1250.

KEIM, D., HAILAT, N., MELHEM, R., ZHU, X.X., LASCU, I., VERON, M., STRAHLER, J. & HANASH, S.M. (1992). Proliferation-related expression of p19/*nm23* nucleoside diphosphate kinase. *J. Clin. Invest.*, **89**, 919-924.

KIMURA, N. & SHIMADA, N. (1988). Membrane associated nucleoside diphosphate kinase from rat liver. *J. Biol. Chem.*, **263**, 4647-4653.

KIMURA, N., SHIMADA, N., NOMURA, K. & WATANABE, K. (1990). Isolation and characterization of a cDNA clone encoding rat nucleoside diphosphate kinase. *J. Biol. Chem.*, **265**, 15744-15749.

LACOMBE, M.-L., SASTRE-GARAU, X., LASCU, I., VONICA, A., WALLET, V., THIERY, J.P. & VERON, M. (1991). Overexpression of nuceloside diphosphate kinase (*nm23*) in solid tumors. *Eur. J. Cancer*, **27**, 1302-1307.

LAKSO, M., STEEG, P.S. & WESTPHAL, H. (1992). Embryonic expression of *nm23* during mouse organogenesis. *Cell Growth Different.*, **3**, 873-879.

LEONE, A., FLATOW, U., KING, C.R., SANDEEN, M.A., MARGULIES, M.K., LIOTTA, L.A. & STEEG, P.S. (1991). Reduced tumor incidence, metastatic potential, and cytokine responsiveness of *nm23* transfected melanoma cells. *Cell*, **65**, 25-35.

LIOTTA, L.A. & STEEG, P.S. (1990). Clues to the function of Nm23 and Awd proteins in development, signal transduction and tumour metastasis provided by studies of Dictyostelium discoideum. *J. Natl Cancer Inst.*, **82**, 1170-1173.

MACDONALD, N.J., ROSA, A.D.L., BENEDICT, M.A., FREIJE, J.M.P., KRUTSCH, H. & STEEG, P.S. (1993). A serine phosphorylation of *nm23*, and not its nucleoside diphosphate kinase activity, correlates with suppression of tumor metastatic potential. *J. Biol. Chem.*, **268**, 25780-25789.

NICKERSON, J.A. & WELLS, W.W. (1984). The microtubule-associated nucleoside diphosphate kinase. *J. Biol. Chem.*, **259**, 11297-11304.

OHTSUKI, K. & YOKOYAMA, M. (1987). Direct activation of guanine nucleotide binding proteins through a high-energy phosphate-transfer by nucleoside diphosphate-kinase. *Biochem. Biophys. Res. Commun.*, **148**, 300-307.

OHTSUKI, K., IKEUCHI, T. & YOKOYAMA, M. (1986). Characterization of nucleoside diphosphate kinase-associated guanine nucleotide-binding proteins from Hela S3 cells. *Biochim. Biophys. Acta*, **882**, 322-330.

OTERO, A.D. (1990). Transphosphorylation and G protein activation. *Biochem. Pharmacol.*, **39**, 1399-1404.

PARHAR, R.S., SHI, Y., ZOU, M., FARID, N.R., ERNST, P. & AL-SEDAIRY, S. (1994). Effects of cytokine mediated modulation of *nm23* expression on the invasion and metastatic behavior of B16F10 melanoma cells. *Int J. Cancer* (in press).

POSTEL, E.H., BERBERICH, S.J., FLINT, S.J. & FERRONE, C.A. (1993). Human c-*myc* transcription factor PuF identified as *nm23*-H2 nucleoside diphosphate kinase, a candidate suppressor of tumor metastasis. *Science*, **261**, 478-480.

ROSENGARD, A.M., KRUTZSCH, H.C., SHEARN, A., BIGGS, J.R., BARKER, E., MARGUILES, I.M.K., KING, C.R., LIOTTA, L.A. & STEEG, P.S. (1989). Reduced Nm23/Awd protein in tumor metastasis and aberrant *Drosophila* development. *Nature*, **342**, 177-180.

SHI, Y., ZOU, M., SCHMIDT, H., JUHASZ, F., STENSKY, V., ROBB, D. & FARID, N.R. (1991). High rates of ras codon 61 mutation in thyroid tumors in an iodide-deficient area. *Cancer Res.*, **51**, 2690-2693.

STAHL, J.A., LEONE, A., ROSENGARD, A.M., PORTER, L., KING, C.R. & STEEG, P.S. (1991). Identification of a second human *nm23* gene, *nm23*-H. *Cancer Res.*, **51**, 445-449.

STEEG, P.S., BEVILACQUA, G., KOPPER, L., THORGEIRSSON, U.P., TALMADGE, J.E., LIOTTA, L.A. & SOBEL, M.E. (1988*a*). Evidence for a novel gene associated with low tumor metastatic potential. *J. Natl Cancer Inst.*, **80**, 200-204.

STEEG, P.S., BEVILACQUA, G., POZZATTI, R., LIOTTA, L.A. & SOBEL, M.E. (1988*b*). Altered expression of *nm23*, a gene associated with low tumor metastatic potential, during adenovirus 2 Ela inhibition of experimental metastasis. *Cancer Res.*, **48**, 6550-6554.

STEEG, P.S., BEVILACQUA, G., ROSENGARD, A.M., SOBEL, M.E., CIOCE, V. & LIOTTA, L. (1989). Altered gene expression in tumor metastasis: the *nm23* gene. In *Cancer Metastasis. Molecular and Cellular Biology, Host Immune Responses and Perspectives for Treatment*, Schirrmacher, V. & Schwartz-Albiez, R (eds) pp. 48-52. Springer: Berlin.

WALLET, V., MUTZEL, R., TROLL, H., BARZU, O., WURSTER, B., VERON, M. & LACOMBE, M.-L. (1990). Dictyostelium nucleotide diphosphate kinase highly homologous to *nm23* and awd proteins involved in mammalian tumor metastasis and *Drosophila* development. *J. Natl Cancer Inst.*, **82**, 1199-1202.

WALTON, G. & GILL, G. (1975). Nucleotide regulation of a eukaryotic protein synthesis initiation complex. *Biochim. Biophys. Acta*, **390**, 231-245.

YAGEL, S., PARHAR, R.S., JEFFREY, J.J. & LALA, P.K. (1988). Normal nonmetastatic human trophoblast cells share *in vitro* invasive properties of malignant cells. *J. Cell Physiol.*, **136**, 455-462.

YOUNG, M.R., OKADA, F., TADA, M., HOSOKAWA, M. & KOBAYASHI, H. (1991). Association of increased tumor cell responsiveness to prostaglandin E2 with more aggressive tumor behaviour. *Invasion Metastasis*, **11**, 48-57.

ZOU, M., SHI, Y., AL-SEDAIRY, S. & FARID, N.R. (1993). High levels of *nm23* gene expression in advanced stage of thyroid carcinomas. *Br. J. Cancer*, **68**, 385-388.